# The association between sonographic enthesitis and radiographic damage in psoriatic arthritis

**DOI:** 10.1186/s13075-017-1399-5

**Published:** 2017-08-15

**Authors:** Ari Polachek, Richard Cook, Vinod Chandran, Dafna D. Gladman, Lihi Eder

**Affiliations:** 1 Centre for Prognostic Studies in the Rheumatic Diseases, Toronto Western Hospital, University of Toronto, 1-412, 399 Bathurst Street, Toronto, ON M5T 2S8 Canada; 2 Department of Statistics and Actuarial Science, University of Waterloo, Waterloo, ON Canada; 3 Department of Medicine and Laboratory Medicine, Institute of Medical Science, University of Toronto, Toronto, ON Canada; 4 Department of Pathobiology, Institute of Medical Science, University of Toronto, Toronto, ON Canada; 5 Centre for Prognosis Studies in the Rheumatic Diseases, Krembil Research Institute, Toronto Western Hospital, Toronto, ON Canada; 6 Center for Prognostic Studies in the Rheumatic Diseases, Toronto Western Hospital, University of Toronto, Toronto, ON Canada; 7 Department of Medicine, University of Toronto, Women’s College Research Institute, Women’s College Hospital, Room 6326, 76 Grenville Street, Toronto, ON M5S 1B2 Canada

**Keywords:** Spondyloarthritis, Pathogenesis, Ultrasound, Steinbrocker, Sacroiliitis, Spondylitis

## Abstract

**Background:**

To examine the association between sonographic enthesitis and the severity of radiographic features of damage in the peripheral and axial joints in psoriatic arthritis (PsA).

**Methods:**

A cross-sectional analysis was conducted in patients with PsA. The MAdrid Sonography Enthesitis Index (MASEI) scoring system was used to quantify the extent of sonographic entheseal abnormalities. Radiographic damage in the peripheral joints and spine was assessed by the modified Steinbrocker score (mSS), Modified New York Criteria for sacroiliitis, and the modified Stoke Ankylosing Spondylitis Spine Score (mSASSS). The association between MASEI and the extent of radiographic damage was assessed using negative binomial and logistic regression. The results were expressed in terms of the regression coefficient estimates and their exponentiated values (e^β^) or odds ratios (OR), and 95% confidence intervals (CI).

**Results:**

Two hundred and twenty three patients were analyzed; 58% were males, with mean ± SD age of 55.9 ± 12.9 years and PsA duration of 16.7 ± 12.4 years. Regression analyses yielded an association between higher MASEI scores (10 units increase) and peripheral joint damage including mSS (e^β^ = 1.42, 95% CI: 1.15, 1.72), joint ankylosis (OR = 1.93, 95% CI: 1.37, 2.72), arthritis mutilans (OR = 1.77, 95% CI: 1.23, 2.54), and periostitis (OR = 1.41, 95% CI: 1.08, 1.84). Similarly, an association was found between higher MASEI scores and axial damage as measured by mSASSS (e^β^ = 2.18, 95% CI: 1.16, 4.09) and sacroiliitis (OR = 1.33, 95% CI: 1.03, 1.72).

**Conclusions:**

The severity of sonographic enthesitis is a potential marker of radiographic peripheral and axial joint damage in PsA.

## Background

Psoriatic arthritis (PsA) is an inflammatory musculoskeletal disease affecting up to a third of psoriasis patients [1, 2]. PsA can affect different locations including the synovial joint, bone, fat pad, bursa, adjacent tendons, and entheses [3]. Enthesitis is a unique feature of the spondyloarthritis (SpA) disease group in general, and of PsA in particular [4, 5]. Clinical enthesitis is a common finding occurring in a third of PsA patients [6, 7].

There are a few imaging modalities that can assist in enthesitis assessment apart from physical examination. Conventional radiography, which usually shows erosions at the enthesis and enthesophytes [8], is limited by its ability to detect mainly chronic irreversible bone damage rather than active inflammation. Another modality is magnetic resonance imaging (MRI) that can demonstrate both active lesions, as entheseal thickness, soft tissue edema, and adjacent bone marrow edema, as well as chronic lesions including erosions and enthesophytes [9]. However, in a recent study that evaluated whole-body MRI, enthesitis was found in only 18% of the patients with PsA and the ability to read images from some locations was technically limited [10]. Musculoskeletal ultrasound (US) assessment is inexpensive, readily available, relatively easy to perform and can evaluate a number of entheseal locations in a short period of time [11]. Several studies found higher sensitivity and specificity of US assessment of the entheses compared with clinical examination [12–15]. Therefore, US has emerged as the preferred modality to assess enthesitis.

The primacy of enthesitis in the pathogenesis of SpA and PsA is a matter of debate. According to the synovio-entheseal model, suggested by McGonagle et al. enthesitis is the initial site of musculoskeletal inflammation in SpA [16]. A few animal model studies support this hypothesis by reporting a link between mechanical stress, enthesitis, and the development of arthritis that is similar to PsA [17–19]. In addition, in a small study that followed 30 psoriasis patients for 3.5 years, the presence of sonographic features of thickness of the quadriceps tendon predicted the development of PsA [20]. However, overall there is limited information about the association between enthesitis and disease outcomes in patients with PsA. We hypothesized that since enthesitis play a role in the pathogenesis of PsA it may serve as a marker of more severe disease outcomes in PsA, alternatively, enthesitis may play a direct role in the development of joint damage in PsA.

Hence, the main objective of the present study was to examine the association between sonographic enthesitis and the severity of radiographic features of damage in the peripheral and axial joints in patients with PsA.

## Methods

### Setting and study population

A cross-sectional study of patients enrolled in the Toronto PsA cohort was conducted. The cohort consists of patients with PsA who are referred to the University of Toronto PsA clinic for the management of their PsA. The patients are enrolled in an ongoing prospective cohort study aimed at assessing prognostic factors in PsA. Each patient is assessed at 6–12-month intervals according to a standard protocol. For the present study, randomly available consecutive patients were recruited for a single ultrasound assessment. The recruitment was completed during two separated time periods: June 2011 to October 2012 (this group of patients participated in our previous study about enthesitis in PsA [21]) and January 2014 to December 2015. Patients were recruited independently of their clinical and radiographic information. All patients satisfied the Classification for Psoriatic Arthritis (CASPAR) criteria [22].

Information collected includes demographics, lifestyle habits including smoking, co-morbid conditions, medications, height, weight, counts of tender swollen and damaged joints, dactylitis, clinical enthesitis assessment according to the SPondyloArthritis Research Consortium Canada (SPARCC) index [22], psoriasis area and severity index (PASI) and the presence of psoriatic nail involvement. The study was approved by the University Health Network Research Ethics Board. All patients signed an informed consent form.

### Ultrasound assessment of enthesitis

A single rheumatologist (LE), who has 7 years of experience in musculoskeletal US and assessment of sonographic enthesitis, performed all US scanning using a MyLab 70 XVG scanner (Esaote, Florence, Italy) equipped with a 6–18 MHz linear transducer (Esaote). Power Doppler settings were standardized with a Doppler frequency of 8.3–10 MHz (depending on body habitus), pulse repetition frequency of 750 Hz, and a wall filter of 2. The following entheseal sites that are part of the MAdrid Sonographic Enthesitis Index (MASEI) scoring system were scanned bilaterally: quadriceps tendon insertion to the patella, patellar tendon insertion to the patella, and tibial tuberosity, Achilles tendon and plantar fascia insertions into the calcaneus, and triceps tendon insertion to the olecranon process. Each tendon was scanned in both longitudinal and transverse planes, and the scan images were stored as short video files for later reading. Each examination took about 20 minutes. The patients were placed in a supine position to assess the patellar and quadriceps entheses. The knee was placed in 30° flexion to assess grayscale abnormalities and in full extension to assess vascularization. Then the patients were placed in a prone position with the feet over the end of the examination table for assessment of the Achilles tendon and plantar fascia entheses. The triceps tendon enthesis was assessed with the elbow flexed to 90° [23].

**Fig. 1:**
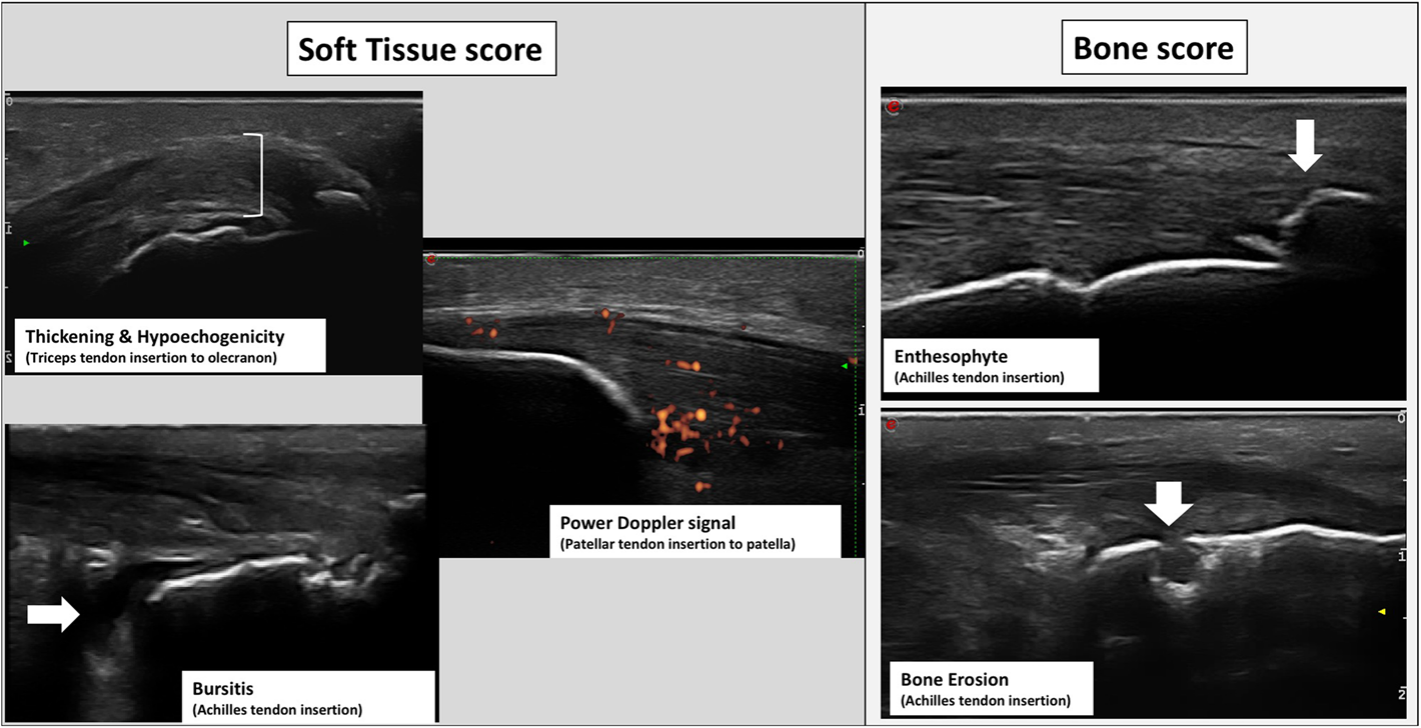
Elementary lesions of enthesitis included in the Madrid Sonography Enthesitis Index (MASEI)

The MASEI was used to generate a global score (range, 0–136) that reflects the severity of entheseal abnormalities in each patient [24]. The enthesopathy US grayscale features of this index include: thickening and structural changes of the tendon insertion, calcific deposits at the tendon insertion, bony changes including erosions and enthesophyte formation as defined by de Miguel et al. [24]. The thickness of the enthesis was measured at the insertion of the deeper tendon margin into the bone in a longitudinal axis. Bursitis was defined as a well circumscribed, localized anechoic or hypoechoic area at the site of an anatomical bursa, which was compressible by the transducer. Vascularization was assessed by the power Doppler within 2 mm of the cortical bone insertion. The sonographic elementary lesions described above are illustrated in Fig. 1. The total MASEI score was further categorized into: (1) bone score, including calcific deposits, erosions and enthesophytes and (2) soft tissue score, including thickening and structural changes of the tendon insertion, bursitis and vascularization. The categorization of sonographic enthesitis scores to chronic/bone score and inflammatory/soft tissue score have been previously used by others [25]. Reading of the US scans was performed independently from the clinical and radiographic data by a single reader (LE). The intra-observer intra-class correlation coefficient for MASEI was 0.8 [23].

### Radiographic joint damage assessment

According to the Toronto PsA clinic protocol, conventional radiography assessment that includes hands, feet, spine, and sacroiliac joints is conducted every 2 years. The reading is performed by at least two rheumatology experts in PsA who were blinded to the US results (DDG, VC).

Peripheral joint damage in 42 joints of the hands and feet was assessed by the modified Steinbrocker score (mSS) [8]. According to this method each joint is scored on a scale ranging from 0 to 4: 0 for normal; 1 for juxta-articular osteopenia or soft tissue swelling; 2 for erosion; 3 for erosion and joint space narrowing, and 4 for total joint destruction. The total mSS is the sum of all scores that are equal or greater than 2 (range 0–168). A score of 1 is not counted towards the total mSS; therefore only definite erosive damage is considered. This method is used to follow radiographic damage in our cohort as it has proved to be valid, reliable, sensitive to change, and feasible to perform in a clinic setting [8]. Additional radiographic features of peripheral joint damage recorded included joint ankylosis, arthritis mutilans that was defined as bone resorption affecting more than 50% of the joint and periostitis that was defined as juxta-articular new bone formation. Axial joint damage was assessed by the modified New York criteria for sacroiliitis [26] and the modified Stoke Ankylosing Spondylitis Spine Score (mSASSS) for spinal damage (score range 0–72) [27].

### Statistical analysis

The analysis was restricted to patients with complete data. Continuous data were described by the mean ± SD and categorical variables were expressed as frequencies and percentages. The association between MASEI score (by 10 units increase, the primary predictor) and the various radiographic features of joint damage (outcomes) was assessed through the use of negative binomial regression models [28] with count responses (mSS and mSASSS) and logistic regression for binary data (ankylosis, mutilans, sacroiliitis, and periostitis).

The initial regression model included only MASEI score as a single co-variate. Multiple regression analyses were then performed in which each regression model xadjusted for age, sex, body mass index (BMI), PsA duration, smoking (past, current, never), and current use of disease-modifying antirheumatic drugs (DMARDs) and biologic medications. A sensitivity analysis was performed with “ever use” instead of “current use” of DMARDs and biologics as model covariates. For negative binomial regression models the effect of MASEI score was expressed through its regression coefficient (β) along with its exponential value (e^β^) and their 95% confidence intervals (CI); the exponentiated value e^β^ is interpreted as the multiplicative effect of 10 units change of MASEI score on the mean response while the other covariates remain unchanged. Odds ratios (OR) were used to express the effect of 10 units increase in the MASEI score in logistic regression models. The effect of a covariate was considered statistically significant if the *P* value was less than 0.05. The statistical analyses were performed using SAS version 9.4 (SAS Institute, Inc., Cary, NC, USA)

## Results

### Baseline characteristics

Two hundred and twenty-three patients were included in the analysis (nine patients were excluded due to missing data). Their mean age was 56 ± 12.9 years (57.9% males) with a mean PsA duration of 16.9 ± 12.4 years (Table 1). Their mean tender and swollen joint counts were 2.5 ± 5.3 and 1.1 ± 3.3, respectively. Clinical enthesitis was found in 13.9% of the patients.

**Table 1:** Characteristics of the study population at the time of the assessment (*N* = 223)

Variable	Value
Demographics:	
Age (years), mean ± SD	56 ± 12.9
Male gender, n (%)	129 (57.9)
PsA duration (years), mean ± SD	16.9 ± 12.4
Psoriasis skin disease duration (years), mean ± SD	28.1 ± 14.5
BMI, mean ± SD	29.8 ± 6.1
Smoking	
Current, n (%)	29 (13)
Past, n (%)	70 (31.4)
Clinical activity measures:	
Tender joints count, mean ± SD	2.5 ± 5.3
Swollen joint count, mean ± SD	1.1 ± 3.3
Clinical enthesitis, n (%)	31 (13.9)
Dactylitis, n (%)	9 (4)
PASI, mean ± SD	2.6 ± 5.8
Nail lesions, n (%)	59 (26.4)
HLA-B*27	34 (15.5)
Radiographic evaluation:	
modified Steinbrocker score	
mean ± SD	18.7 ± 33.6
median (25^th^, 75^th^ percentile)	4 (0, 20)
Arthritis mutilans, n (%)	20 (9)
Joint ankylosis, n (%)	26 (11.7)
Periostitis, n (%)	49 (22.2)
mSASSS,	
mean ± SD	1.7 ± 7.2
median (25^th^, 75^th^ percentile)	0 (0, 0)
Sacroiliitis, n (%),	82 (37.1)
Sonographic evaluation:	
MASEI, mean ± SD	15.7 ± 12.6
Treatment:	
DMARDs – current use, n (%)	102 (45.7)
DMARDs – ever use, n (%)	181 (81.2)
Biologics – current use, n (%)	119 (53.4)
Biologics – ever use, n (%)	130 (58.3)

*PsA* psoriatic arthritis, *BMI* body mass index*,* BOD*PASI* psoriasis area and severity index, HLA-B*27 human leukocyte antigen B*27, *mSASSS* modified Stoke Ankylosing Spondylitis Spine Score, *MASEI* MAdrid Sonography Enthesitis Index, *DMARDs* disease-modifying antirheumatic drugs

The mean MASEI score was 15.6 ± 12.6 and the mean mSS was 18.7 ± 33.6. With respect to axial radiographic damage, the mean mSASSS was 1.7 ± 7.2 and 37.1% of the patients had sacroiliitis.

### The association between MASEI and peripheral radiographic damage

The results of the statistical analyses assessing the association between MASEI score and the various measures of peripheral joint damage are shown in Table 2. Multiple regression analysis yielded an association between MASEI score and mSS so that a 10-unit increase in MASEI was associated with a 42% higher mSS (e^β^ = 1.42, 95% CI 1.15, 1.72). Both MASEI bone and soft tissue subscores were independently associated with mSS. In addition, an association was found between a MASEI score and additional outcomes of peripheral joint damage (Table 3). A higher MASEI score was also associated with ankylosis such that a 10-unit increase in MASEI almost doubled the odds of ankylosis (OR = 1.93, 95% CI 1.37, 2.72). Similarly, both MASEI bone and soft tissue subscores were associated with this outcome. Finally, an association was found between MASEI score (10 units increase) and arthritis mutilans (OR 1.77, 95% CI 1.23, 2.54) and periostitis (OR 1.41, 95% CI 1.08, 1.84). When the components of the MASEI score were examined separately, only MASEI bone score was associated with these outcomes.

**Table 2:** The association between MASEI and modified Steinbroker score - negative binomial regression

Variable^*^	Univariate regression			Multiple regression^**^		
	estimate (SE)	exp^(Estimate)^ (95% CI)	*P* value	estimate (SE)	exp^(Estimate)^ (95% CI)	*P* value
MASEI – total	0.48 (0.11)	1.61 (1.30, 1.99)	<0.001	0.35 (0.10)	1.42 (1.15, 1.73)	<0.001
MASEI – bone score	0.86 (0.19)	2.36 (1.63, 3.38)	<0.001	0.64 (0.18)	1.89 (1.33, 2.69)	<0.001
MASEI – soft tissue score	0.63 (0.20)	1.88 (1.26, 2.77)	0.002	0.37 (0.18)	1.44 (1.03, 2.05)	0.03

*MASEI* MAdrid Sonography Enthesitis Index

^*^10 units increase in MASEI

^**^Each model was adjusted for age, sex, PsA duration, BMI, smoking, current use of DMARDs and biologics

**Table 3:** The association between MASEI score and features of radiographic damage – Logistic regression analysis

Variable*	Univariate regression		Multiple regression^**^	
	OR (95% CI)	*P* value	OR (95% CI)	*P* value
Joint ankylosis				
MASEI total	2.05 (1.52 2.75)	<0.001	1.93 (1.37, 2.72)	<0.001
MASEI – bone score	3.49 (2.09, 5.85)	<0.001	3.24 (1.75, 6.01)	<0.001
MASEI – soft tissue score	2.59 (1.55, 4.31)	<0.001	2.96 (1.36, 4.22)	0.003
Arthritis mutilans				
MASEI total	1.96 (1.43, 2.69)	<0.001	1.77 (1.23, 2.54)	0.002
MASEI – bone score	3.94 (2.24, 6.93)	<0.001	3.77 (1.84, 7.70)	<0.001
MASEI – soft tissue score	2.00 (1.15, 3.50)	0.014	1.71 (0.90, 3.26)	0.102
Periostitis				
MASEI total	1.48 (1.17, 1.88)	0.001	1.41 (1.08, 1.84)	0.013
MASEI – bone score	2.05 (1.35, 3.11)	<0.001	1.88 (1.17, 3.02)	0.009
MASEI – soft tissue score	1.65 (1.07, 2.53)	0.022	1.52 (0.96, 2.41)	0.07
Sacroiliitis				
MASEI total	1.36 (1.10, 1.70)	0.005	1.33 (1.03, 1.72)	0.027
MASEI – bone score	2.12 (1.42, 3.16)	<0.001	2.24 (1.40, 3.61)	<0.001
MASEI – soft tissue score	1.23 (0.84, 1.83)	0.285	1.10 (0.71, 1.70)	0.664

*MASEI* MAdrid Sonography Enthesitis Index

^*^10 units increase in MASEI

^**^Each model was adjusted for age, sex, PsA duration, BMI, smoking, current use of DMARDs and biologics

### The association between MASEI and axial radiographic damage

The results of the regression models assessing the association between MASEI score and the various measures of axial joint damage are shown in Table 3 and 4. Multiple regression analyses yielded an association between a higher MASEI score (10 units increase) and mSASSS (e^β^ 2.18, 95% CI 1.16, 4.09). Both the MASEI bone and soft tissue subscores were associated with higher mSASSS scores. In addition, an association was found between a higher MASEI score (10 units increase) and the presence of sacroiliitis (OR 1.33, 95% CI 1.03, 1.72). MASEI bone subscore was associated with sacroiliitis, but the MASEI soft tissue subscore was not.

**Table 4:** The association between MASEI and mSASSS – negative binomial regression

Variable^*^	Univariate model			Multivariable model^**^		
	estimate (SE)	exp^(Estimate)^ (95% CI)	*P* value	estimate (SE)	exp^(Estimate)^ (95% CI)	*P* value
MASEI – total	0.65 (0.25)	1.92 (1.17, 3.09)	0.009	0.78 (0.32)	2.18 (1.16, 4.09)	0.02
MASEI – bone score	1.23 (0.42)	3.42 (1.52, 7.69)	0.003	1.51 (0.51)	4.52 (1.68, 12.18)	0.003
MASEI – soft tissue score	1.04 (0.50)	2.89 (1.06, 7.61)	0.04	0.70 (0.69)	2.01 (0.52, 7.77)	0.31

*MASEI* MAdrid Sonography Enthesitis Index

^*^10 units increase in MASEI

^**^Each model was adjusted for age, sex, PsA duration, BMI, smoking, current use of DMARDs and Biologics

### Sensitivity analysis

In a sensitivity analysis, the model covariates “current use of DMARDs” and “current use of biologics” were replaced by “ever use of DMARDs” and “ever use of biologics”. No significant changes in the results were observed (data not shown).

## Discussion

It was Ball in 1971 who primarily set the foundations for the significance of enthesitis in SpA, by suggesting that the enthesis is centrally affected in ankylosing spondylitis (AS) patients, while the synovial joint is the main target of the inflammatory involvement in rheumatoid arthritis (RA) patients [29]. A few decades later, McGonagle and colleagues contributed significantly to the understanding of enthesitis as a key feature in SpA [16, 30]. Over the years, this idea was recognized by different organizations as the Assessment of Spondyloarthritis International Group (ASAS) that included it in their classification criteria for both axial and peripheral SpA and by the Group for Research and Assessment of Psoriasis and Psoriatic Arthritis (GRAPPA) who included it in the stem requirements of the CASPAR criteria [21, 31, 32].

The current study provides novel data regarding the association between enthesitis and features of severity in patients with PsA. The study found that a higher MASEI score, which reflects more severe enthesitis, is associated with severity of peripheral radiographic joint damage as measured by mSS. Furthermore, the severity of enthesitis was associated with proliferative and erosive features of joint damage including joint ankylosis, arthritis mutilans and periostitis. Additionally, a higher enthesitis score was associated with features of axial radiographic damage, including syndesmophytes and sacroiliitis.

According to the synovio-entheseal complex model, enthesitis is the primary lesion that triggers the related inflammation in the adjacent synovial joint [16]. This hypothesis is supported by a study that compared early SpA to early RA patients and demonstrated that knee synovitis was associated with entheseal abnormalities only in the SpA group [33]. Additional studies from the same group found associations between enthesitis and various features of SpA as dactylitis, tenosynovitis, arthritis mutilans, distal interphalangeal joint involvement, and psoriatic nail disease [34]. Supporting the importance of enthesitis in peripheral musculoskeletal inflammation in PsA, other studies that compared PsA and RA patients found that sonographic entheseal abnormalities in the fingers were identified only in the PsA group [4, 5]. Finally, a recent study in transgenic TNFα mice demonstrated that the initial signs of inflammation were found at the entheses and subsequently new bone formation appeared at entheseal sites and correlated with the degree of inflammation [19]. In accordance with these findings, our study found an association between the extent of enthesitis and peripheral joint damage.

However, there are studies that could not confirm the specific link between enthesitis and SpA [35, 36]. Paramarta et al. found the same prevalence of entheseal involvement in SpA and RA patients [35]. Ibrahim et al. found similar sonographic entheseal scores in patients with PsA and RA [36]. However, the significantly older age of the RA group in their study may have accounted for the higher entheseal score in this group leading to the similarity in scores. Overall, the discrepancies between the different studies can be attributed to the lack of standardization manifesting with diversity in several aspects including the type of the patients, the sample size, disease manifestations, enthesitis sites scanned, sonographic indices, and imaging equipment.

Unfortunately, there are only a few longitudinal studies that may address the controversy regarding the primacy of enthesitis in the pathogenesis of SpA [20, 37]. Tinazzi et al. found that thickness of the quadriceps tendon predicated the development of clinical PsA in a small cohort of psoriasis patients [20]. El-Miedany et al. evaluated 126 psoriasis patients by clinical, radiological and sonographic measures over a period of 1 year [37]. However, this study did not differentiate between the two pathologies as it found that both sonographic enthesitis and synovitis at baseline were predictors of joint damage in new-onset PsA patients. Finally, a recent study in 41 psoriasis patients found that arthralgia and baseline MRI synovitis in the hand joints predicted the development of clinical PsA after 1 year of follow-up [38]. The detection of periarticular inflammation in only 4% of the patients raises a question regarding the reliability of this tool for enthesitis evaluation.

Enthesitis is a widespread condition that can involve the peripheral as well as the axial skeleton. The location of inflammation in spondylitis is at the entheses, where the ligaments attach to the vertebrae [39]. Several studies showed an association between clinical enthesitis [measured by the Maastricht Ankylosing Spondylitis Enthesis Score (MASES)] and both higher disease activity [measured by the Bath Ankylosing Spondylitis Disease Activity Index (BASDAI)] and worse functional status [40, 41]. In addition, Muche et al. reported that enthesitis in the sacroiliac region, as detected by MRI, was more common in advanced sacroiliitis [42]. Our study found an association between sonographic enthesitis and axial damage, including both spondylitis and sacroiliitis. In contrast to our study, Alcalde et al. did not find a correlation between sonographic enthesitis and radiographic sacroiliitis [43]. However, their study is not entirely comparable to ours for several reasons: first, the study population included AS and not PsA patients, second, the sample size was much smaller including only 44 patients and lastly, the sonographic enthesitis index included less entheseal sites and did not include Power Doppler vascularization assessment.

The agreement between sonographic and clinical assessment of enthesitis is an area of interest in rheumatology due to the limited specificity and sensitivity of the latter method [15]. A few studies that compared these two modalities found low to moderate agreement [43, 44]. Recently, van der Ven et al. reported that adding US evaluation in psoriasis patients who had entheseal tenderness on clinical examination reduced the rate of enthesitis by 64% [45]. In this regard, it should be mentioned that the MASEI score includes entheseal lesions that represent irreversible damage from prior active enthesitis, such as enthesophytes and erosions, in addition to active lesions such as vascularization. Thus, the sonographic score may be high in patients who sustained entheseal damage from prior enthesitis who may be in remission at the time of assessment.

This study has several limitations. First, the association between sonographic enthesitis in sites located adjacent to large joints and radiographic damage of small joints in the hands and feet is indirect and may not suggest a causal link. Identifying enthesitis in the small joints is a difficult task, which may explain why there is no such enthesitis index. In addition, radiographic damage in large joints is less common and hence there is no standardized damage index for these sites. Thus, although the study cannot support a direct causal link between enthesitis and the development of peripheral or axial joint damage, the strong association observed between the extent of enthesitis and a number of features of joint damage in the peripheral and axial joints suggest that enthesitis may be a marker of more severe PsA phenotypes. An additional limitation of the study includes its cross-sectional nature that does not allow making causal inferences. Furthermore, we assessed the associations between enthesitis at a single time point and radiographic damage that has accumulated over time. However, MASEI score includes a bone damage subcategory which reflects irreversible entheseal bone damage (e.g., enthesophytes and erosions) that has accumulated over time. Furthermore, all regression analyses showed that these chronic bone lesions were more strongly associated with radiographic joint damage than the soft tissue lesions. Lastly, treatments may have modified the relationship as we controlled for treatment only at the time of assessment, although it is expected that effective treatment for enthesitis, such as biologics, would have weakened the association. However, our sensitivity analyses that included the use of biologics and DMARDs at any point during the disease did not significantly modify the results.

This study has several strengths. To our knowledge, this is the largest study thus far that explored the association between sonographic enthesitis and radiographic joint damage in PsA. The use of sonographic enthesitis as a primary predictor improved the accuracy of assessing this important feature over physical examination [15]. In addition, the PsA cohort in this study is well phenotyped, which enables to control for multiple confounders.

## Conclusions

The results of the study suggest that the severity of sonographic enthesitis is a potential marker of radiographic peripheral and axial joint damage in PsA. The association was found with both erosive and proliferative bone lesions. These findings raise the question of whether enthesitis has a role in the pathogenesis of articular damage in PsA. Further longitudinal studies in early PsA patients are required in order to establish the precise cause and effect relationships between enthesitis, synovitis, and joint damage.

## Abbreviations

AS: Ankylosing spondylitis
ASAS: Assessment of Spondyloarthritis International Group
BMI: Body mass index
CASPAR: Classification for psoriatic arthritis
CI: Confidence interval
DMARDs: Disease-modifying antirheumatic drugs
GRAPPA: Group for Research and Assessment of Psoriasis and Psoriatic Arthritis
MASEI: MAdrid Sonography Enthesitis Index
MRI: Magnetic resonance imaging
mSASSS: modified Stoke Ankylosing Spondylitis Spine Score
mSS: modified Steinbrocker score
OR: Odds ratios
PASI: Psoriasis area and severity index
PsA: Psoriatic arthritis
RA: Rheumatoid arthritis
SpA: Spondyloarthritis
SPARCC: SPondyloArthritis Research Consortium Canada
US: Ultrasound
