# Correction to: The association between sonographic enthesitis and radiographic damage in psoriatic arthritis

**DOI:** 10.1186/s13075-019-1814-1

**Published:** 2019-01-14

**Authors:** Ari Polachek, Richard Cook, Vinod Chandran, Dafna D. Gladman, Lihi Eder

**Affiliations:** 1 Centre for Prognostic Studies in the Rheumatic Diseases, Toronto Western Hospital, University of Toronto, 1-412, 399 Bathurst Street, Toronto, ON M5T 2S8 Canada; 2 Department of Statistics and Actuarial Science, University of Waterloo, Waterloo, ON Canada; 3 Department of Medicine and Laboratory Medicine, Institute of Medical Science, University of Toronto, Toronto, ON Canada; 4 Department of Pathobiology, Institute of Medical Science, University of Toronto, Toronto, ON Canada; 5 Centre for Prognosis Studies in the Rheumatic Diseases, Krembil Research Institute, Toronto Western Hospital, Toronto, ON Canada; 6 Center for Prognostic Studies in the Rheumatic Diseases, Toronto Western Hospital, University of Toronto, Toronto, ON Canada; 7 Department of Medicine, University of Toronto, Women’s College Research Institute, Women’s College Hospital, Room 6326, 76 Grenville Street, Toronto, ON M5S 1B2 Canada; 8 Department of Rheumatology, Tel Aviv Sourasky Medical Center, Sackler Faculty of Medicine, Tel Aviv University, Tel Aviv, Israel

**Correction to: Arthritis Res Ther**

**https://doi.org/10.1186/s13075-017-1399-5**

Following publication of the original article [1], the authors reported an error in the affiliation of Ari Polachek. The correct affiliation of Dr. Polochek is:

^1^Centre for Prognostic Studies in the Rheumatic Diseases, Toronto Western Hospital, University of Toronto, 1-412, 399 Bathurst Street, Toronto, ON M5T 2S8, Canada.

^8^Department of Rheumatology, Tel Aviv Sourasky Medical Center, Sackler Faculty of Medicine, Tel Aviv University, Tel Aviv, Israel.
